# The Adjuvanticity of an *O. volvulus*-Derived r*Ov*-ASP-1 Protein in Mice Using Sequential Vaccinations and in Non-Human Primates

**DOI:** 10.1371/journal.pone.0037019

**Published:** 2012-05-17

**Authors:** Jing Wang, Nancy Tricoche, Lanying Du, Meredith Hunter, Bin Zhan, Gaddam Goud, Elizabeth S. Didier, Jing Liu, Lu Lu, Preston A. Marx, Shibo Jiang, Sara Lustigman

**Affiliations:** 1 Lindsley F. Kimball Research Institute, New York Blood Center, New York, New York, United States of America; 2 Division of Microbiology, Tulane National Primate Research Center, Covington, Louisiana, United States of America; 3 Department of Microbiology, Immunology and Tropical Medicine, George Washington University and the Sabin Vaccine Institute, Washington, D.C., United States of America; London School of Hygiene and Tropical Medicine, United Kingdom

## Abstract

Adjuvants potentiate antigen-specific protective immune responses and can be key elements promoting vaccine effectiveness. We previously reported that the *Onchocerca volvulus* recombinant protein r*Ov*-ASP-1 can induce activation and maturation of naïve human DCs and therefore could be used as an innate adjuvant to promote balanced Th1 and Th2 responses to bystander vaccine antigens in mice. With a few vaccine antigens, it also promoted a Th1-biased response based on pronounced induction of Th1-associated IgG2a and IgG2b antibody responses and the upregulated production of Th1 cytokines, including IL-2, IFN-γ, TNF-α and IL-6. However, because it is a protein, the r*Ov*-ASP-1 adjuvant may also induce anti-self-antibodies. Therefore, it was important to verify that the host responses to self will not affect the adjuvanticity of r*Ov*-ASP-1 when it is used in subsequent vaccinations with the same or different vaccine antigens. In this study, we have established r*Ov*-ASP-1's adjuvanticity in mice during the course of two sequential vaccinations using two vaccine model systems: the receptor-binding domain (RBD) of SARS-CoV spike protein and a commercial influenza virus hemagglutinin (HA) vaccine comprised of three virus strains. Moreover, the adjuvanticity of r*Ov*-ASP-1 was retained with an efficacy similar to that obtained when it was used for a first vaccination, even though a high level of anti-r*Ov*-ASP-1 antibodies was present in the sera of mice before the administration of the second vaccine. To further demonstrate its utility as an adjuvant for human use, we also immunized non-human primates (NHPs) with RBD plus r*Ov*-ASP-1 and showed that r*Ov*-ASP-1 could induce high titres of functional and protective anti-RBD antibody responses in NHPs. Notably, the r*Ov*-ASP-1 adjuvant did not induce high titer antibodies against self in NHPs. Thus, the present study provided a sound scientific foundation for future strategies in the development of this novel protein adjuvant.

## Introduction

The use of an adjuvant is a key element in promoting vaccine effectiveness because it can stimulate the immune system and accelerate, prolong, or enhance antigen-specific immune responses, even when used in combination with weak vaccine antigens [1]. Adjuvants have been used in vaccines since the early 20th Century following more than 100 years of research. In the U.S., Alum remains the sole FDA-approved adjuvant for general use of vaccines [2]. However, few adjuvants have been licensed for use around the world [3]. No new adjuvants have been approved in the United States since the 1930s, and only recently has the European Heads of Medicines Agencies licensed the MF59, AS03 and AS04 adjuvants for use with the Fluad®, Fendrix™ and Cervarix™ defined vaccines, respectively [4]. Cervarix™ which uses AS04, a combination of aluminum hydroxide and monophosphoryl lipid A (MPL), in its formulation was also licensed by FDA on October 16, 2009, to prevent cervical cancer caused by human papillomavirus types 16 and 18. Adjuvants are important in guiding the type of adaptive response that is induced after vaccination and that is most effective against incoming infections. The development of novel adjuvants that stimulate discrete subsets of immune cells, in particular, cytotoxic T-lymphocytes (CTL), is required to unleash the full potential of new vaccines and immunotherapy strategies [5]. Although tremendous progress has been made in the development of many vaccine platforms, including DNA-based vaccines, recombinant subunit vaccines, viruses and conjugates, the absence of safe and effective adjuvants impedes the clinical development of such new generations of vaccines. Interest in developing new adjuvants has increased significantly over the past decade, as highlighted by the following issues [6], [7]: 1) the inability of traditional approaches to develop successful vaccines against “difficult” organisms such as HIV and HCV; 2) the emergence of epidemics or outbreaks of new infectious diseases with high mortality, especially those causing serious threats to public health and socioeconomic stability worldwide (e.g., SARS, Ebola, West Nile, Dengue, pandemic flu and nvCJD); 3) the re-emergence of “old” infections like tuberculosis; 4) the continuing spread of antibiotic-resistant bacteria; and 5) the increased threat of bioterrorism. Therefore, the molecular design of potently adjuvanted vaccines that would enhance antigen uptake *in vivo* and potentially also simplify their adjuvant requirements would be highly desirable [8].

**Figure 1:**
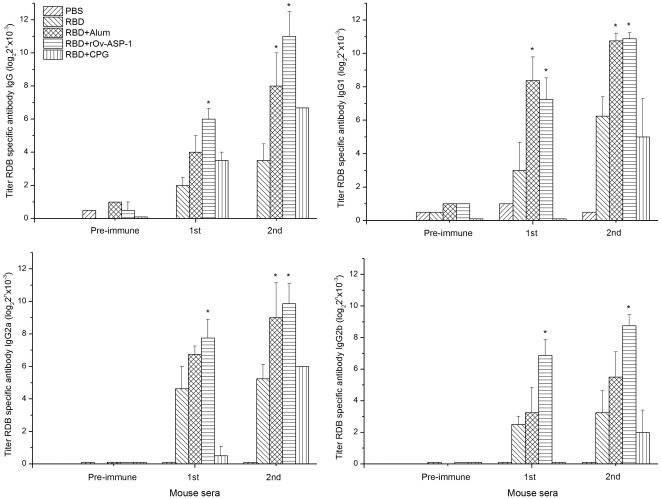
Anti-RBD antibody responses in vaccinated mice. RBD-specific responses in mice immunized with recombinant SARS-CoV RBD in the presence of the r*Ov*-ASP-1, Alum or CpG. Titer of RBD- specific IgG and IgG subtypes was detected by ELISA using sera from mice before (pre-immune) and 10 days after each vaccination. * indicates significant difference (*p*<0.05) among multiple comparisons; in particular between mice that were immunized in the presence of r*Ov*-ASP-1 or control adjuvants vs. no adjuvant.

There are many reports showing that helminth-derived molecules have potent regulatory or stimulatory effects on the immune system of their mammalian hosts (reviewed in [9], [10]–[21]). Some of these molecules were shown to contain pathogen associated molecular pattern that bind to endocytic-pattern recognition receptors on antigen presenting cells (APCs). Three helminth products have also been reported to act as adjuvants in experimental vaccine models. Proteins secreted by adult *Nippostrongylus brasiliensis* (NES) induced strong Th2 responses in mice immunized with hen egg lysozyme [22]. NES actively matured dendritic cells (DC) and selectively up-regulated CD86 and OX40L, together with IL-6 production, while blocking IL-12p70 responsiveness in a manner consistent with Th2 generation *in vivo* [23]. Similarly, lacto-*N*-fucopentaose III (LNFPIII), a carbohydrate found on the surface of the eggs of a human parasite, *Schistosoma mansoni*, acted as a Th2 adjuvant for human serum albumin when injected intranasally, subcutaneously or intraperitonealy into mice [24]. It functions as an innate Th2 promoter via its action on murine DCs. Its ability to drive DC2 maturation was shown to be dependent on signaling via toll-like receptor 4 (TLR4) [25]. Finally, when co-administered with an inactivated anti-influenza vaccine in both young and aged mice, a 19 aa synthetic peptide (GK-1) from *Taenia crassiceps* cysticerci has induced increased levels of anti-influenza antibodies in aged mice, both before and after infection, reduced the local inflammation that accompanied influenza vaccination itself, and favored virus clearance after infection in both young and aged mice [26].

**Figure 2:**
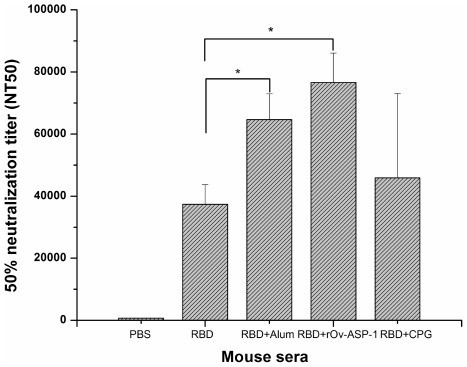
Neutralizing antibody titers induced in vaccinated mice. Neutralization of SARS pseudovirus infection by mouse antisera from each rRBD immunization group 10 days after second boost. Infection of 293T/ACE2 cells by SARS pseudovirus was determined in the presence of antisera at a series of 2-fold dilutions, and 50% neutralization titer (NT_50_) was calculated for each sample. * indicates significant difference (*p*<0.05).

A recombinant *Onchocerca volvulus* activation-associated protein-1, *rOv*-ASP-1, has been shown to be a novel protein adjuvant that can increase specific immune responses in mice, both humoral and cellular responses, against recombinant protein or peptide-based antigens when formulated in aqueous mixtures [14], [27], [28], [29]. We have previously suggested that these effects are probably attained through the cellular activation of APCs such as dendritic cells via TLR-2 and TLR-4 [27]. The *rOv*-ASP-1 adjuvant is able to induce balanced Th2 and Th1-associated IgG1 and IgG2a antibodies to proteins, polypeptides and small peptides. With a few antigens such as rgp41, RBD and HBsAg, it also promoted a putative Th1-biased response based on pronounced induction of Th1-associated IgG2a and IgG2b antibody responses and/or a significantly upregulated production of Th1 cytokines, including IL-2, IFN-γ, TNF-α, and IL-6 [14], [27], [29], [30] . Its ability to augment Th1-associated antibody responses was further demonstrated in studies using three commercial inactivated vaccines against hemorrhagic fever with renal syndrome, flu and Rabies [29]. Moreover, in a novel recombinant configuration, r*Ov*-ASP-1 fused to 3 copies of the highly conserved extracellular domain of the H5N1 influenza M2 protein sequence was able to induce high levels of M2e-specific IgG, IgG1, IgG2a providing strong cross-protection from a lethal challenge with 3LD_50_ or 10 LD_50_ of H5N1 viruses of different clades (clade 1: VN/1194, or clade 2.3.4: SZ/406H [28]. In this study, we further demonstrated the adjuvanticity of r*Ov*-ASP-1 in sequential vaccines in mice and also confirmed its ability to be a potent innate adjuvant in NHPs.

**Table 1:** Cytokine responses to rRBD and its CD4^+^ and CD8^+^ epitopes in vaccinated mice.

Cytokine(pg/ml)	Vaccine Groups			
	RBD+PBS	RBD+r*O*v-ASP-1	RBD+Alum	RBD+CPG
**Responses to rRBD**				
IL-2	33±6	173±8	129±13	17±5
IL-6	20±12	61±12	107±11	31±6
IL-10	9±2	1,175±91	1,858±137	220±45
IFN-γ	20±1	594±2	659±216	881±60
TNF-α	31±8	165±15	141±37	86±75
IL-17A	8±3	27±1	79±24	168±95
**Responses to N50 (CD8^+^ epitope)**				
IL-2	22±4	164±15	177±23	36±26
IL-6	5±1	55±10	79±2	0±0
IL-10	3±1	545±221	929±6	30±7
IFN-γ	3±0	428±25	550±17	290±176
TNF-α	14±1	102±4	120±13	40±49
IL-17A	4±1	29±22	19±1	5±7
**Responses to N60 (CD4^+^ epitope)**				
IL-2	36±6	165±31	183±12	48±24
IL-6	15±3	70±4	88±15	18±3
IL-10	6±3	887±337	1,076±63	67±1
IFN-γ	3±1	726±139	505±8	170±28
TNF-α	14±0	111±14	138±2	27±26
IL-17A	7±1	28±6	43±33	20±19

## Results

### The r*Ov*-ASP-1 adjuvant enhances humoral and cellular responses after immunization with rRBD of SARS-CoV in mice

To evaluate the adjuvant activity of r*Ov*-ASP-1, mice were immunized with SARS-CoV rRBD in the presence or absence of r*Ov*-ASP-1 or with Alum or CpG for comparison. We previously reported that r*Ov*-ASP-1 could effectively induce a mixed, but Th1-skewed immune response against rS and rRBD in immunized mice [27]. As shown in Figure 1, the titers of RBD-specific IgG, IgG1, IgG2a and IgG2b antibodies increase after the first or second boost immunization in the sera of mice immunized with rRBD plus r*Ov*-ASP-1, which were all significantly higher than in mice immunized with rRBD alone. The IgG and IgG2a responses in mice vaccinated with rRBD in the presence of Alum were significantly higher only after the second boost; only the IgG1 response was significantly higher after the first boost. The anti-rRBD IgG response was significantly higher in the presence of CpG only after the second boost when compared to mice vaccinated only with rRBD (1∶102,400 vs. 1∶11,314). Notably, the r*Ov*-ASP-1 augmented IgG antibody response to rRBD was almost 4 times higher than the Alum vaccination group (64,000 vs. 16,000) and 5.7 times higher than the CpG vaccination group (64,000 vs. 11,200) already after the first boost. Further comparison of the r*Ov*-ASP-1 and the Alum induced rRBD antibody responses after the second boost revealed similar levels of IgG1 (1,884,544 vs. 1,722,156); two-fold higher level of IgG2a (935,763 vs. 512,000) as well as ten times higher level of IgG2b (430,538 vs. 45,255) endpoint titers in the r*Ov*-ASP-1 vaccine group. Comparison of the r*Ov*-ASP-1 and the CpG induced rRBD antibody responses after the second boost revealed a 27 fold increase in IgG1 (1,884,544 vs. 68,000), 14.6 fold increase in IgG2a (935,763 vs. 64,000), and 215 fold increase in IgG2b (430,538 vs. 2,000) endpoint titers. Each adjuvant/rRBD model performed differently depending on the adjuvant, e.g., a skewed Th2 response with Alum (IgG1/IgG2a = 3.3), but a mixed Th1/Th2 response with CpG (IgG2a/IgG1 = 0.94) and r*Ov*-ASP-1(IgG2a/IgG1 = 2), with a predominance of Th1-associated antibodies (when both IgG2a and IgG2b are taken into account) with r*Ov*-ASP-1. These results further supported our previous studies showing that r*Ov*-ASP-1 can induce a more balanced antibody response with some bias towards a skewed Th1-associated antibody response than other adjuvants used with the same bystander antigen such as Alum or CpG (this study) or the MLP plus TDM adjuvant [14], [27], [29].

**Figure 3:**
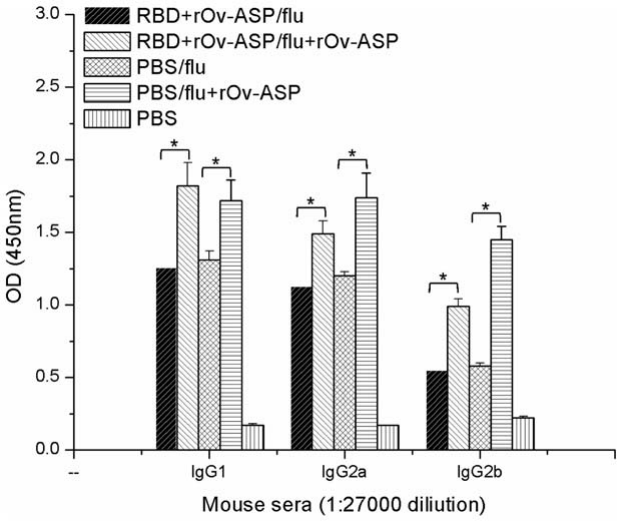
Anti- HAs antibody responses in vaccinated mice. Antibody responses to HAs in mice vaccinated previously with rRBD in combination with the r*Ov*-ASP-1 adjuvant were compared to those generated in mice after primary immunization with the HA vaccine in the presence or absence of the r*Ov*-ASP-1 adjuvant. The IgG isotype responses in individual mouse against influenza virus HAs from three virus strains were tested by ELISA at 1∶27,000 dilutions. * indicates significant difference (*p*<0.05) among multiple comparisons; in particular between mice that were immunized in the presence of r*Ov*-ASP-1 vs. no adjuvant.

To further evaluate whether the induced IgG antibodies could neutralize infection of SARS-CoV *in vitro* (Fig. 2), we tested the antisera from the 10-day post-second boost immunization and found that mice immunized with rRBD in the presence of r*Ov*-ASP-1 contained a very high titer of neutralizing antibodies against infection by SARS-CoV pseudovirus (NT_50_ = 1∶76,592), which was not significantly different than in neutralizing antibodies found in sera from mice immunized with rRBD plus the Alum adjuvant (NT_50_ = 1∶64,666).

The subclass of immunoglobulin induced after immunization is an indirect measure of the relative contribution of Th1-type cytokines vs.Th2-type cytokines. In this study, the data from the CBA analyses showed that the levels of Th1- and Th2-type cytokine secretion were significantly higher in mice immunized with rRBD+r*Ov*-ASP-1 or Alum than in those who were immunized with rRBD alone (Table 1). The r*Ov*-ASP-1 induced the production of Type I proinflammatory cytokines (IL-2, IFN-γ, TNF-α, IL-17A and IL-6) to the same extent as Alum, as well as the Th2/regulatory cytokines IL-6 and IL-10. There was no significant recall induction of the Th2 IL-4 or IL-5 cytokines by rRBD (data not shown). Notably, the responses to rRBD when formulated with CpG are in comparison more IFN-γ and IL-17A dominant with diminished IL-2, TNF-α, IL-10 and IL-6 responses. We found that the variation between individual mice was very low [31], [32], and therefore we are confident that the results obtained using the pooled spleens are a good representation of what would have been the outcome if we had used individual mice.

**Table 2:** Titers of anti-HA antibody response in rRBD+ r*O*v-ASP-1 vaccinated mice and naïve mice after vaccination with an influenza vaccine or an influenza vaccine in combination with r*O*v-ASP-1.

	End point titer 7 days post boost		
Vaccination group	IgG1	IgG2a	IgG2b
(RBD+r*O*v-ASP-1)+flu	607,500	486,000	162,000
(RBD+r*O*v-ASP-1)+(flu+r*O*v-ASP-1)	1,458,000	243,000	243,000
PBS+flu	729,000	486,000	54,000
PBS+(flu+r*O*v-ASP-1)	1,458,000	729,000	243,000

Distinct from the previous studies are the responses to two RBD-specific peptides; N50 (CD8^+^ T cell epitope) and N60 (CD4^+^ T cell epitope) (Table 1). Interestingly, the cytokine levels in the culture supernatants of the murine splenocytes when stimulated with either N50 or N60 were similar to those produced by stimulation with the full length rRBD in mice vaccinated with rRBD+r*Ov*-ASP-1, thus further confirming the ability of r*Ov*-ASP-1 to elicit RBD-specific CD8^+^ and CD4^+^ cellular responses in the tested vaccine formulation.

**Table 3:** RBD-specific IgG responses in NHPs immunized with rRBD in the presence of r*Ov*-ASP-1 or CpG adjuvant.

Immunization	Anti-RBD IgG titer					
rRBD +	50 µg	50 µg	100 µg	100 µg	500 µg	
	r*O*v-ASP-1	r*O*v-ASP-1	r*O*v-ASP-1	r*O*v-ASP-1	CpG	PBS
Prebleed	<100	<100	<100	<100	<100	<100
7 days post prime	400	<100	400	<100	<100	<100
7 days post 1st boost	800	200	1,600	6,400	6,400	<100
7 days post 2nd boost	400	3,200	3,200	3,200	51,200	<200
7 days post 3nd boost	3,200	3,200	6,400	6,400	102,400	200

### The r*Ov*-ASP-1 adjuvant enhances immune responses in a sequential influenza vaccine

Using a mouse model in which we first immunized mice with rRBD of SARS-CoV, we evaluated the efficacy of r*Ov*-ASP-1 adjuvanticity in a sequential vaccination in which we used the HAs of three influenza viruses (A/Brisbane/59/2007, IVR-148 (H1N1); A/Uruguay/716/2007, NYMC X-175C (H3N2) (an A/Brisbane/10/2007-like virus); and B/Brishbane/60/2008) as the model antigens. Naïve BALB/c mice or mice ten weeks after immunization with rRBD in the presence of r*Ov*-ASP-1 were immunized with HAs of influenza viruses in the absence or presence of yeast expressed r*Ov*-ASP-1 (100 µg/mice). The mice that were previously immunized with rRBD in the presence of r*Ov*-ASP-1 had endpoint total anti-r*Ov*-ASP-1 antibody titers of 1∶256,000–1∶512,000 at the time of the priming with the second vaccine. As shown in Table 2, similar IgG1 and IgG2a humoral immune responses against the influenza viruses were induced in the mice vaccinated previously with rRBD plus r*Ov*-ASP-1 adjuvant and those administered with PBS only. Moreover, the IgG2b was higher in the group that got the sequential vaccine (162,000 vs. 54,000). The anti-HA IgG1 and IgG2b antibody responses were much higher in mice that were immunized with the HAs vaccine in the presence of r*Ov*-ASP-1 adjuvant than those immunized in the absence of r*Ov*-ASP-1 adjuvant (IgG1: 1,458,000 vs. 607,000 or 729,000; IgG2b: 243,000 vs. 162,000 or 54,000). All levels of IgG isotype responses in mice when the flu vaccine was formulated with rOv-ASP-1 were statistically higher than when mice were immunized with no adjuvant (*p*>0.05; Fig. 3). There was no significant difference in the IgG1 and IgG2a responses if the flu vaccine was given to naïve mice or to mice that were previously immunized with another vaccine; rRBD of SARS-CoV+r*Ov*-ASP-1.

**Figure 4:**
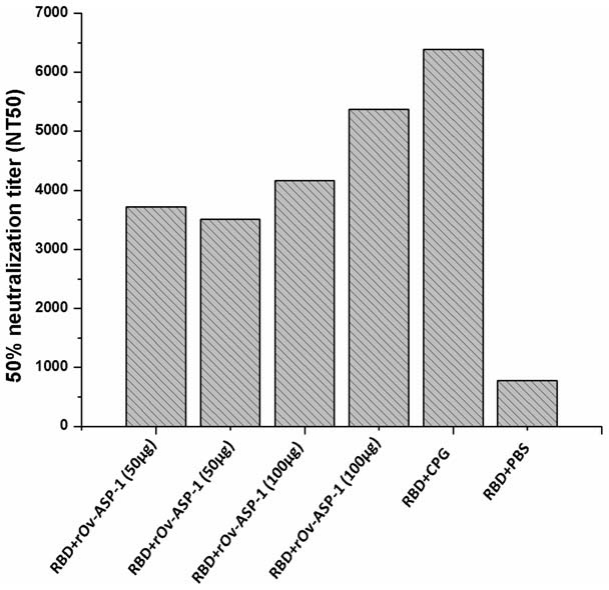
Neutralizing antibody titers induced in vaccinated NHPs. Neutralizing antibody titers induced in NHPs immunized with recombinant SARS-CoV RBD in the presence of 50 µg (N = 2) or 100 µg (N = 2) of r*Ov*-ASP-1 or 500 µg of the CpG adjuvant. Neutralizing antibody titers against SARS pseudovirus using 293T/ACE cells were determined in the presence of antisera from each of the RBD-immunized NHPs. The data are presented as 50% neutralization titer (NT_50_).

### The r*Ov*-ASP-1 as an adjuvant enhances humoral and cellular responses in NHPs vaccinated with rRBD of SARS-CoV

The adjuvanticity of r*Ov*-ASP-1 was evaluated in a rhesus macaque immunization model using rRBD as the target antigen of the SARS-CoV vaccine. As shown in Table 3, all of the NHPs vaccinated with rRBD protein plus 50 µg (n = 2), 100 µg r*Ov*-ASP-1 (n = 2) or 500 µg CpG (n = 1) as the adjuvant developed RBD-specific IgG antibody response with increasing antibody level after each boost. RBD-specific antibodies were not detected in the pre-immune sera of the vaccinated rhesus macaques or the rhesus macaques injected with RBD+PBS control (n = 1). Immunization with rRBD plus an optimized quantity of the CpG (500 µg) as the adjuvant as used in other vaccine studies [33], [34] was the most effective for the induction of RBD-specific antibodies, with endpoint IgG titer of 102,400 after third boost.

Although this was a limited pilot experiment using only two concentrations of the *E. coli* expressed r*Ov*-ASP-1 adjuvant for the formulation (50 µg, 100 µg), which was two or four times the amount used in our mouse model experiments (25 µg), we clearly show that immunization with 50 or 100 µg of the r*Ov*-ASP-1 adjuvant exhibited a dose-dependent efficacy in the induction of RBD-specific antibodies in the two macaques per group; endpoint IgG titers of 3,200 (50 µg ) or 6,400 (100 µg). Notably, the anti-r*Ov*-ASP-1 antibody response titers to the adjuvant itself were lower in the NHPs than we might have expected based on the data in mice; the range in all the 4 immunized macaque monkeys was: 1∶100–1∶800 after first boost, 1∶800–1∶1,600 after second boost and 1∶200–1;1600 after the third boost.

Although the anti-RBD endpoint tiers were much higher in the NHP that was immunized with CpG, the differences in the NT_50_ titers were less prominent. Sera from all rhesus macaques vaccinated with the rRBD+adjuvant formulation effectively neutralized the infection of SARS pseudovirus in 293T cells expressing the receptor ACE2 (ACE2/293T) with NT_50_ values of 1∶3,500–1∶6,392. As shown in Figure 4, sera from macaques immunized with rRBD protein plus 100 µg r*Ov*-ASP-1 induced a slightly higher titer of neutralizing antibody than the 50 µg r*Ov*-ASP-1 group; 4,167/5,376 vs. 3,724/3,509. The macaques that were immunized with CpG had a higher NT_50_ titer at 1∶6,392.

In an *in vitro* study, as described above, we showed that mice immunized with rRBD+r*Ov*-ASP-1 had a strong cytokine production *ex vivo* when stimulated with rRBD protein or RBD-specific peptides N50 and N60. Notably, only a significant TNF-α response was obtained in NHPs that were immunized with rRBD in the presence of r*Ov*-ASP-1 or CpG *ex vivo* when PBMCs were stimulated with 5 µg/ml of rRBD (Table 4). The cytokine response was similar regardless of the amount of r*Ov*-ASP-1 used as the adjuvant. All other secreted cytokines, such as IFN-γ, IL-2, IL-4, IL-5 and IL-6 were negligible.

**Table 4:** TNF-α response in immunized NHPs.

Immunization	pg/ml
rRBD+PBS	152
rRBD+r*Ov*-ASP-1 (50 µg)	1,056
rRBD+r*Ov*-ASP-1 (50 µg)	1,292
rRBD+r*Ov*-ASP-1 (100 µg)	1,350
rRBD+r*Ov*-ASP-1 (100 µg)	771
rRBD+CpG (500 µg)	1,129

## Discussion

Vaccines remain the most effective means of preventing or eradicating infectious diseases, and there are ongoing efforts to apply active immunization approaches to prevent and treat autoimmune diseases and cancer [35], [36]. Adjuvants potentiate antigen-specific immune responses and can be a key element of vaccine effectiveness [1], [37], [38].

Adjuvants can be broadly separated into two classes based on their principal mechanism of action: vaccine delivery systems and immunostimulation. Vaccine delivery systems are generally particulate and function mainly to target associated antigens into APCs, e.g., emulsions, microparticles, iscoms, and liposomes [6], [39]. Immunostimulatory adjuvants contain residues that are recognized by receptors on APCs, such as TLRs, which play an important role in the innate recognition of pathogens by DCs, and thus directly activate innate immune responses. These adjuvants are now regarded as the most effective means by which an adjuvant-antigen complex can target APCs [40]. Adjuvants targeting multiple innate immune receptors may prove to be the most effective adjuvants, as they may induce different arms of the immune responses in the host. A number of microbial products, including bacterial LPS, peptidoglycan, dsRNA, muramyl peptides, CpG, flagellin and microbial proteins, were shown to act as vaccine adjuvants [41]–[45]. Some of these immunomodulators could skew acquired immune responses towards a Th1-type immune response. Adjuvants can also be classified according to their capacity to stimulate either innate or adaptive immunity based on significant differences in their cellular receptors and mechanisms of action [46]. As previously noted, Alum is the only adjuvant licensed in the U.S. for general use in humans. Yet, Alum is not effective in stimulating Th1 and/or cytotoxic T cell responses to a number of pathogens and is therefore limited in its applications, in particular for new-generation vaccines [47]. Some of the adjuvants being developed in clinical testing include MPL, the saponin derivative QS-21, CpG, flagellin, and combinations of some of these adjuvants [40]–[51].

One of the major concerns regarding the use of immunostimulatory adjuvants in humans is the possible increased risk of autoimmune diseases due to targeting pattern recognition receptors by such adjuvants. However, the recombinant *Ov*-ASP-1 adjuvant we studied corresponds to a secreted filarial protein, which is presented as an antigen in the *O. volvulus* exposed or infected individuals in Africa. There is no evidence to show that this secreted filarial protein could induce autoimmune disease in the infected patients, thus excluding such a concern.

Polarized Th1-type immunity can be achieved by the addition of complete Freund's adjuvant and CpG DNA to an antigen [52]–[54]. On the other hand, Th2 antibody responses can be induced by the Alum or incomplete Freund's adjuvant, as indicated by increased IgG1 relative to IgG2a [53]–[55]. However, in situations where both Th1 and Th2 responses are required for protection, the choice of one regimen over another might be counter effective. This has led to additional research for alternative adjuvants or adjuvant combinations that promote balanced mixed Th1/Th2 responses. The present study clearly demonstrated that r*Ov*-ASP-1 could effectively induce mixed RBD- or HA-specific Th1/Th2 antibody associated responses, when used as an adjuvant with recombinant subunit vaccine or as an addition to a commercial Flu vaccine (FLUVIRIN; using 20% of the dose recommended for human use).

Since the r*Ov*-ASP-1 adjuvant is a protein, it is potentially processed and presented to the immune system and subsequently induces antibodies against self. Therefore, concerns might be raised whether preexisting anti-r*Ov*-ASP-1 antibodies may suppress its adjuvanticity when it is used in subsequent vaccine formulations. Previously we demonstrated that antibody response to the adjuvant itself did not hindered the development of OVA, rS or rRBD antigen-specific antibody responses after each boost; in all cases the responses in the presence of r*Ov*-ASP-1 were more elevated than those in the presence of MPL+TMP or Alum adjuvants. Our present results further confirmed that antibodies induced against the adjuvant had no impact on its ability to induce immune responses against bystander antigens when used as an adjuvant in a sequential vaccine when two vaccine model systems were used: the RBD of SARS-CoV spike protein and a commercial influenza virus HA vaccine comprised of three virus strains. Even though a high level of anti-r*Ov*-ASP-1 antibodies (1∶256,000–1∶512,000) was present in the sera of mice before the administration of the second vaccine, the adjuvanticity of r*Ov*-ASP-1 was retained with efficacy similar to that obtained when it was used as an adjuvant in a first vaccine immunization of naïve mice (Table 2 and Figure 3); no difference in the IgG titers was observed between the two vaccine groups, mice pre-immunized with rRBD+r*Ov*-ASP-1 or PBS. Thus, we confirmed that the preexisting anti-r*Ov*-ASP-1 antibodies induced by a previously administrated r*Ov*-ASP-1-based vaccine do not have a significant effect on the adjuvanticity of r*Ov*-ASP-1 when formulated in a subsequent vaccine. Notably, immunization of NHPs with r*Ov*-ASP-1 did not induce high titers of anti-self-antibodies, for reasons that are not yet clear. Future studies will be needed to further validate that even these reasonably low antibody responses to the adjuvant have no impact on subsequent use of this adjuvant with other vaccines also in NHPs.

The pilot immunogenicity studies in the NHPs have provided us with extremely valuable “Proof of Principal” information in an outbred primate model. Firstly, no adverse reactions were observed at the site of the immunizations, indicating the safety of rOv-ASP-1 as an adjuvant. Secondly, using two concentration of the r*Ov*-ASP-1 adjuvant, 50 or 100 µg, and rRBD as the vaccine antigen, we were able to induce after three immunizations high titers of neutralizing antibodies (1∶3,500–1∶6,392) that much exceed what is needed for protection against SARS-CoV infection *in vivo* (>1∶500) [56]. Moreover, our studies have shown that r*Ov*-ASP-1 was able to support the induction of functional antibodies against a pathogen in NHPs, thus clearing the way for its future development for human vaccines as well. Thus, this pilot NHP study is a definitive step for demonstrating relevance of r*Ov*-ASP-1 for human vaccine formulations, even though more studies will be needed to find the optimized amount of the adjuvant in vaccine formulations, which will specify the putative starting adjuvant dose for future clinical trials of r*Ov*-ASP-1-based vaccines. Interestingly, we did not see augmented recall RBD specific cellular responses except TNF-á. Therefore, future studies will be needed to validate the adjuvanticity of r*Ov*-ASP-1 in primates from the point of its ability to augment the desired types of cellular responses are associated with protective immunity against possibly other pathogens, duration of immunity and the potential to establish T cell memory. In summary, the present studies have advanced our confidence that the r*Ov*-ASP-1 protein adjuvant can be further developed for human use, particularly when strong functional balanced antibody responses are needed against the pathogens.

Importantly, the r*Ov*-ASP-1 that was used to immunize mice previously immunized with RBD+r*Ov*-ASP-1 or the naïve mice with the HA vaccine was expressed in the yeast. Having a functional yeast expressed adjuvant will support future development of a scalable process for the downstream manufacture of r*Ov*-ASP-1, including the development of a series of critical biochemical and biophysical assays for in-process, release and stability testing, which will result in a high-yield reproducible production process and a stable product that is highly potent and stimulates the desired antibody and cellular responses to co-administered vaccine antigens in NHPs for further analyses and for future clinical trials in humans.

## Materials and Methods

### Animals and Ethics Statement

Animal protocols were approved by the Institutional Animal Care and Use Committee at the New York Blood Center (mice, Protocols #255 and #194) and the Tulane National Primate Research Center (NHPs, Protocol #P0052). The Tulane National Primate Research Center TNPRC is a USDA-inspected and Association for Assessment and Accreditation of Laboratory Animal Care International (AAALAC)-approved facility, and has an Animal Welfare Assurance on file with the Office of Laboratory Animal Welfare. All animal studies were carried out in strict accordance with the recommendations of the American Veterinary Medical Association (AVMA) Guidelines and the approved protocols. The animals were handled delicately. Any treatment was done with extreme care and professionalism to avoid any unnecessary discomfort for the animals.

Forty- two female BALB/c mice aged 4–6 weeks and six purpose-bred adult male Indian-origin rhesus macaques aged 6–10 years were used in this study. Animal housing and environmental conditions met all applicable standards. Blood was collected retro-orbitally for mice and from the femoral vein using the Sarstedt S-Monovette system for NHPs. The animals were anesthetized using Ketamine (0.1 ml/kg IM), which ameliorate any suffering of the animals during the blood draw or immunization. The NHP protocol also included a full CBC and Chem20 profile, which were taken at each blood drawing, with results falling within normal levels. Their body weight was frequently monitored and physical examinations were performed regularly by the attending veterinarian.

### Expression of the recombinant Ov-ASP-1 protein in *E.* coli and yeast

The r*Ov*-ASP-1 protein was expressed as a histidine-tagged protein in *Escherichia coli* and purified as previously described [27]. The purified r*Ov*-ASP-1 was tested negative in a *Limulus* amoebocyte lysate assay. A quantitative LPS testing by Cambrex BioScience showed that purified r*Ov*-ASP-1 contained <0.25 endotoxin units per milligram of the protein (25 pg endotoxin/mg), and we considered it as an LPS-free stock.

In addition, we expressed r*Ov*-ASP-1 in yeast. Yeast codon optimized *Ov*-*asp*-1 cDNA sequence (AF020586) without the region encoding the N-terminal signal peptide was cloned into the *Pichia* expression vector pPICZαA (Invitrogen) using the *Eco*RI and *Xba*I restriction sites. The recombinant plasmid was linearized with *Sac*I digestion and transformed into *Pichia pastoris* X33 strain as described previously [57]. The positive transformants were screened on Zeocin-resistant YPD plates, and the highest expression clone was selected by scale up culturing. The expression of r*Ov*-ASP-1 with 6× His and c-myc tag at C-terminus was induced with 0.5% methanol and scaled up in 10 liters fermentation as described previously [58].

The r*Ov*-ASP-1 was purified from the fermentation culture with SP Sepharose Fast Flow exchange chromatography as described previously [58]. Briefly, the fermentation supernatant was filtered through a 0.22 µm membrane and the pH was adjusted to 4.8 by adding glacial acetic acid. The positively charged r*Ov*-ASP-1 was captured onto cation SP Sepharose FF column and eluted with 50 mM sodium acetate, pH 4.8 containing 200 mM NaCl. The eluted r*Ov*-ASP-1 pool was then purified and buffer exchanged using gel filtration chromatography (Sephadex G-25 Fine) into PBS, pH 7.2 [28], [57]. The product was tested for its adjuvanticity in mice using OVA as the model antigen as previously described [27] and it was established that 100 µg yeast-expressed r*Ov*-ASP-1 per mouse was as effective as 25 µg per mice of the *E. coli* expressed rOv-ASP-1; both eliciting after two immunizations end point anti-OVA IgG titers of 1∶25,600 (data not shown). Thus, the *E. coli* or the yeast expressed r*Ov*-ASP-1 at their optimal quantities can be used intermittently with assurance.

### Immunization procedures in mice and NHPs

Mice were vaccinated subcutaneously with rRBD protein purified from culture supernatant of transfected human embryonic kidney cell-line 293T (HEK293T) (ATCC, VA) according to the previously described protocol [31], [59], [60] using 20 µg/mouse of the protein mixed in aqueous solution with the *E. Coli*-expressed optimized quantity of r*Ov*-ASP-1 (25 µg/mouse) in 200 µl. As adjuvant controls we immunized mice with rRBD mixed with Imject Alum (40 mg/mL Aluminum hydroxide+40 mg/mL magnesium hydroxide, Thermo Scientific) diluted 1∶1 with the vaccine antigen in a total volume of 200 µl per mouse or with rRBD mixed with 50 µg CpG-ODN1826 (InvivoGen, San Diego CA) in a total volume of 200 µl per mouse. Mice immunized with rRBD in PBS were used as the negative control. The mice were boosted twice 3 weeks apart with 10 µg/mouse of rRBD protein in PBS, *rOv*-ASP-1, Imject Alum or CpG. All mice were bled retro-orbitally under anaesthesia prior to immunizations and 10 days post injections. Sera were stored at −80°C.

To test the adjuvanticity of *rOv*-ASP-1 in a sequential vaccine model, we immunized intramuscularly naïve mice or mice that were previously immunized with the rRBD+r*Ov*-ASP-1 with a commercial flu vaccine (100 µl/mouse) containing 3 µg of influenza virus hemagglutinin (HA) from each of the following 3 viruses: A/Brisbane/59/2007, IVR-148 (H1N1); A/Uruguay/716/2007, NYMC X-175C (H3N2) (an A/Brisbane/10/2007-like virus); and B/Brishbane/60/2008 (FLUVIRIN, Novartis). The immunization was done in the presence or absence of the yeast-expressed optimized quantity of r*Ov*-ASP-1 (100 µg/mouse). The mice were boosted once with the same dose of vaccines three weeks later. All mice were bled prior to immunization and 7 days post immunization as described above and sera were stored at −80°C.

Rhesus macaques were vaccinated subcutaneously with rRBD protein (50 µg) with either 50 µg (n = 2) or 100 µg (n = 2) of *E. coli*-expressed r*Ov*-ASP-1, CpG-C-ISS-ODN C274 (500 µg; n = 1) (donated by Dynavax Technologies Corporation, Berkeley, CA) or PBS (n = 1). They were boosted with the same dose at 1-, 2- and 6-month intervals. The macaques were bled before immunization and 7 days post-immunization. Sera were collected for serological testing, and PBMCs were purified for *in vitro* assays. A baseline bleed was also provided.

### Measurement of antigen-specific antibody responses

ELISA was used to detect specific antibody responses induced in the vaccinated mouse or NHP. Briefly, 96-well micro titer plates (Costar) were coated with rRBD (1 µg/ml), r*Ov*-ASP-1 (1 µg/ml) or HAs (2.5 µg/ml) at 4°C overnight. The plates were blocked with 2% non-fat milk in PBS-Tween (0.05%) for 2 h at 37°C. After washing the plates six times with PBS-T, serial diluted sera in binding buffer (1% non-fat milk in PBS-T) were added into each well in duplicate and incubated for 1 h at 37°C. Bound antibodies were detected with HRP-conjugated goat anti-mouse IgG, IgG1, IgG2a or IgG2b (Molecular Probes-Invitrogen) (1∶2000 dilution) or mouse anti-monkey IgG-HRP (clone SB108a, Southern Biotech) (diluted 1∶1000) in binding buffer. After incubation for 1 h at 37°C, plates were washed and TMB substrate (KPL) was added, and the reaction was stopped by the addition of 2 N H_2_SO_4_. Absorbance at 450 nm was measured using SpectraMax 190 (Molecular Devices, Sunnyvale, CA). End point titers were defined as the highest dilutions giving an *A*_450_ measurement of 0.1. This cutoff value represents the value of mean optical density (OD) plus 2 standard deviations (SD) of 10 normal mouse serum samples tested at 1∶100 dilutions or the 6 pre-bleeds from the normal NHP serum samples also tested at 1∶100 dilutions.

### SARS pseudovirus neutralization assay

The neutralization assay against SARS pseudovirus infection was performed as previously described [61]. In brief, plasmid DNA encoding SARS-CoV spike protein and a plasmid DNA encoding Env-defective, luciferase-expressing HIV-1 genome (pNL4-3.luc.RE) were co-transfected into HEK293T cells (ATTC, GA). Culture supernatant containing SARS pseudovirus was collected at 72 h and used for single-cycle infection *in vitro*. The SARS pseudovirus was incubated in the presence or absence of serially diluted antisera from vaccinated mice or NHPs for 1 h at 37°C. Subsequently, the antisera-virus mixtures were added to 293T cells expressing the SARS receptor angiotensin-converting enzyme 2 (293T/ACE2) in 96-well plates, and the infection was allowed to proceed for 48 h, followed by lysing the infected cells using cell lysis buffer included in the luciferase kit (Promega). Aliquots of cell lysates were transferred to 96-well Costar flat-bottom luminometer plates (Corning Costar), followed by addition of luciferase substrate (Promega). Relative light units were determined immediately in the Ultra 384 luminometer (Tecan). The neutralization of SARS pseudovirus is presented as 50% neutralizing antibody titer (NT_50_) [60].

### Cytokine assays

Th1/Th2/Th17 cytokine assay was used to estimate the corresponding cytokine production *ex vivo* from splenocytes of the vaccinated mice. Briefly, splenocytes were harvested from the immunized mice and resuspended in RPMI 1640 medium (Invitrogen, Carlsbad, CA) supplemented with 10% FBS (Hyclone Laboratories, Inc.), 2 mM L-glutamine, 10 mM HEPES, and Antibiotic-Antimycotic (Invitrogen, Carlsbad, CA). The cells were plated at 4×10^5^ cells into 96-well U-bottom culture plates for *in vitro* stimulation with 5.0 µg of rRBD, or with the SARS-CoV RBD-specific N50 (CD8^+^ T cell epitope) or N60 (CD4^+^ T cell epitope) peptide [31]; a concentration that was pre-determined to be optimal. Cells were stimulated with or without PMA (10 ng/ml) plus ionomycin (1 µg/ml) as the positive and negative controls, respectively. The plates were incubated at 37°C for 72 h, and the secreted cytokines were quantified from the culture supernatants using the Mouse Th1/Th2/Th17 BD Cytometric Bead Array Kit (BD Biosciences) according to the manufacture's protocols. Theoretical limit of detection data is IL-2 = 0.1 pg/ml; IL-4 = 0.03 pg/ml; IL-6 = 1.4 pg/ml; IL-10 = 16.8 pg/ml; TNF-α = 0.9 pg/ml, INF-γ = 0.5 pg/ml; and IL-17A = 0.8 pg/ml.

Detection of the Th1/Th2 cytokine production in the vaccinated NHPs was done using similar protocol as above with some modifications. PBMCs were isolated following a Ficoll-Hypaque density gradient (Sigma). Single-cell suspensions were then stimulated at 4×10^5^ cells with 5 µg of rRBD, N50 or N60 peptides, or PMA (10 ng/ml) plus ionomycin (1 µg/ml) for positive control and culture media alone for negative control. The cells were stimulated for 5 days, and cytokines were quantified using the Non-Human Primate Th1/Th2 BD Cytometric Bead Array Kit (BD Biosciences) according to the manufacture's protocols.

### Statistical analysis

Results are expressed as mean ± SEM. The data were analyzed using GraphPad version 5.01 for Windows (GraphPad software). Unpaired two-tailed Student's *t* test or Mann-Whitney test was used to compare means between different groups. One-way ANOVA with Bonferroni post-test was considered appropriate for multiple comparisons. *P* value less than 0.05 was considered significant.
